# Reduced Activity of Nitrate Reductase Under Heavy Metal Cadmium Stress in Rice: An *in silico* Answer

**DOI:** 10.3389/fpls.2018.01948

**Published:** 2019-01-15

**Authors:** Prerna Singh, Indra Singh, Kavita Shah

**Affiliations:** ^1^Department of Biochemistry, Faculty of Science, Banaras Hindu University, Varanasi, India; ^2^Faculty of Science, School of Biotechnology, Banaras Hindu University, Varanasi, India; ^3^Institute of Environment and Sustainable Development, Banaras Hindu University, Varanasi, India

**Keywords:** cadmium, nitrate reductase, nitrate, nitrite, rice

## Abstract

Cadmium is a well known toxic heavy metal, which has various detrimental effects on plant system. In plants an important enzyme involved in the production of nitric oxide, nitrate reductase, is also affected by cadmium toxicity. According to many studies cadmium has an inhibitory effect on nitrate reductase activity. Similar effect of cadmium was found in our study where an inhibitory effect of cadmium on nitrate reductase activity was noted. However, the mechanism behind this inhibition has not been explored. With the help of homology, 3-D structure of rice-nitrate reductase is modeled in this study. Its binding with nitrate, nitrite and cadmium metal *in silico* has been explored. The bonds formed between the enzyme-substrate complex, enzyme-cadmium and differences in interactions in presence of cadmium has been studied in detail. The present study should help in understanding the modeled structure of rice-nitrate reductase in 3-D which may in turn guide enzyme related studies *in silico*. The present study also provides an insight as to how cadmium interacts with nitrate reductase to alter the enzyme activity.

## Introduction

**Figure 1:**
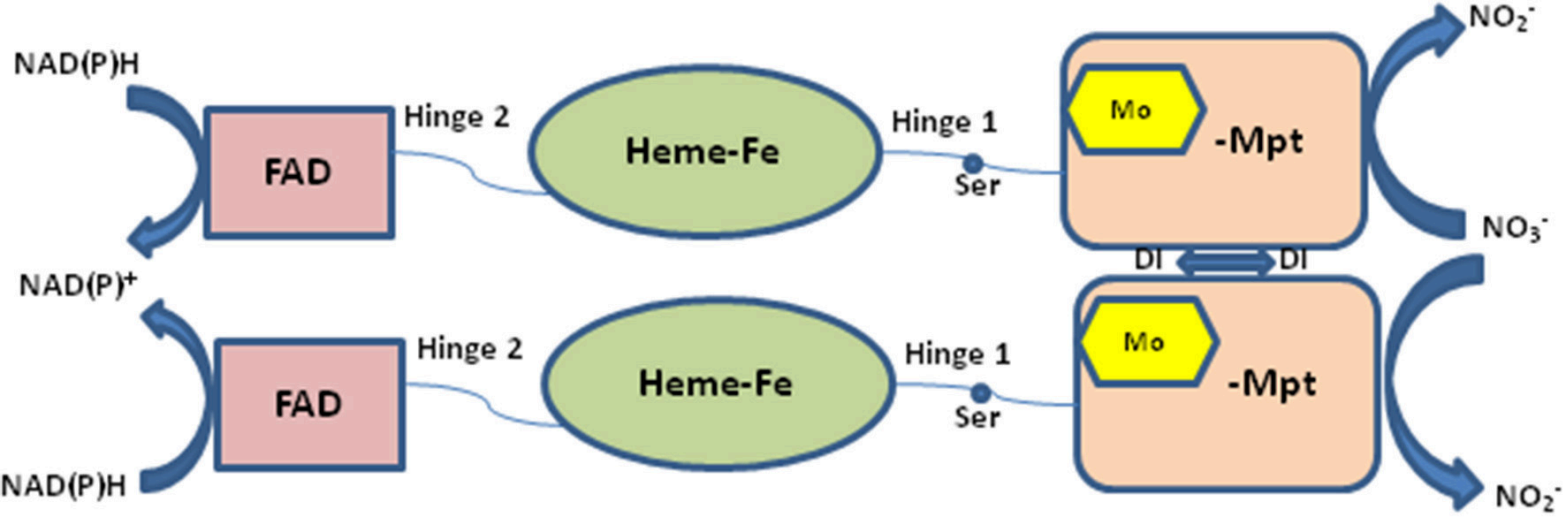
Structure of nitrate reductase enzyme. FAD, flavine adenine dinucleotide; Ser, serine; Mo, molybdenum; Mpt, molybdopterin; NAD(P)H, nicotinamide adenine dinucleotide phosphate.

Nitrate reductase (NR EC 1.7.1.1-3) is a molybdoenzyme involved in the production of nitric oxide in the plant system. NR contains molybdenum as cofactor (Moco) and comes under the category of enzymes that take part in two-electron transfer reactions in all carbon, nitrogen, and sulfur cycles (Hille, 1996). All eukaryotic enzymes containing molybdenum have a molybdenum (Mo) atom attached to two sulfur atoms (ene-dithiolate) of molybdopterin (a pyranopterin derivative). The molybdopterin contains a highly conserved structural core which is present in nearly all Mo containing enzymes except for nitrogenase (Fischer et al., 2005). In its active form eukaryotic NR is present as a homodimer and the enzyme is dependent on Moco for its dimerization (Campbell, 2001). There are three separate domains in a NR monomer: the Mo center, the Fe-heme of the cytochrome b5 domain, and a C-terminal domain associated with a flavin adenine nucleotide (FAD) cofactor (Figure 1). The Mo containing domain is responsible for the dimerization of NR, this Mo containing domain is subdivided into N-terminal (Moco binding) and C-terminal (mediates dimerization) which was identified in the crystal structure of the homologous sulfite oxidase (SO). NR contains two linker region named hinge 1 and hinge 2. Hinge 1 distinguishes the cytochrome b5 domain from the dimerization domain and hinge 2 separates cytochrome domain and the FAD domain from each other (Figure 1). There is a regulatory serine residue on hinge 1 in plants which inhibits NR upon its phosphorylation (Kaiser and Huber, 2001; MacKintosh and Meek, 2001). Reduction of nitrate to nitrite by NR occurs via three steps: first- a reductive half-reaction where electrons are transferred from nicotinamide adenine dinucleotide phosphate (NAD(P)H) to reduce FAD, second- electron transfer through the intermediate cytochrome b5 domain, and third an oxidative half-reaction where electrons are transferred from Mo center to nitrate which converts to nitrite (Skipper et al., 2001). The classical views suggested that NR directly reduces nitrite to produce nitric oxide (Dean and Harper, 1988; Klepper, 1990; Yamasaki et al., 1999; Yamasaki and Sakihama, 2000; Morot-Gaudry-Talarmain et al., 2002; Meyer et al., 2005; Medina-Andrés et al., 2015) but recent studies have brought this fact into light that there exists another Mo-enzyme, NO-forming nitrite reductase (NOFNiR), distinct from NR which reduces nitrite to NO with the participation of NR to provide the electrons (Chamizo-Ampudia et al., 2016, 2017). Cadmium is a toxic heavy metal which is introduced in the environment through various sources and possesses detrimental effects on plant system such as membrane damage, interference in electron transport chain, activation and inhibition of various enzymes and changes at DNA level (Shah et al., 2001). Oxidative stress is a common consequence of cadmium toxicity which occurs due to unrestricted formation and accumulation of free oxygen radicals like H_2_O_2_, OH, ${\text{O}}_{2}^{.-}$, etc (Di Toppi and Gabbrielli, 1999; Singh and Shah, 2012; Shah et al., 2013). Activity of various antioxidant enzymes such as catalase, peroxidase, superoxide dismutase is also altered under cadmium toxicity (Hegedüs et al., 2001; Shah et al., 2001, 2013). Enzyme NR is also affected by cadmium toxicity. Many reports suggest that Cd has an inhibitory effect on NR activity (Gouia et al., 2000; Faizan et al., 2011; Hayat et al., 2011). Similar effect of Cd was found in our present study where a decrease in NR activity was found upon Cd stress on rice seedlings. Giving positive feedback to the speculation that NR is involved in nitric oxide production, in our previous study also a decrease in nitric oxide content was noted in rice seedlings when subjected to Cd stress (Singh and Shah, 2014). With the knowledge of complete rice genome (Ohyanagi et al., 2006) it is expected that the 3-D structures of important rice proteins will be available in the database online. Surprisingly, no crystallographic structure could be obtained or is reported for rice NR. With the help of homology modeling, 3-D structure of rice-NR (OsNR) is modeled in this study. Binding of OsNR with nitrate and cadmium metal *in silico* has been explored. We have also studied the interaction of OsNR with nitrite. A detailed study of interactions between enzyme-substrate complex and cadmium and the alterations in the bonds formed in presence of cadmium is carried out *in silico*. The bonds formed between the enzyme-substrate complex, enzyme-cadmium and differences in interactions in presence of cadmium has been studied in detail *in silico*. The present study should help in understanding the modeled structure of rice-NR in 3-D which may in turn direct enzyme related studies *in silico*. The present study also provides an insight as to how cadmium interacts with NR to alter the enzyme activity.

## Methods

### Plant Material and Stress Conditions

Rice seeds from *cv*. HUR 3022 were surface sterilized with 0.1% sodium hypochlorite solution and imbibed in water for 24 h. Seeds were germinated in petriplates for 3 days, seedlings were transferred and raised in sand cultures for next 7 days in plastic pots saturated with either Hoagland nutrient solution (Hoagland and Arnon, 1950) which served as controls or nutrient solution supplemented with 50 μM Cd(NO_3_)_2_. The choice of 50 μM Cd(NO_3_)_2_ in this study was based upon the seed viability test performed under increasing concentrations of Cd(NO_3_)_2_ and reported earlier by our lab (Shah, 1995). Seedlings were maintained in the growth chamber at 28 ± 1°C, 80% relative humidity and 12-h light/dark cycle (irradiance 40–50 μmol m^−2^ s^−1^) as described earlier (Shah et al., 2001). Pots were maintained at field saturation capacity and irrigation was done when required. Seedlings were uprooted from 1, 4, 7 (days counted after transfer to sand culture with treatment) day old rice seedlings, the roots and shoots were separated and experiments were performed in triplicate.

### Determination of Nitrate Reductase Activity

NR activity was measured according to Jin et al. (2011). Briefly, leaves from rice seedlings were excised and placed in each test tube. 5 mL of assay solution comprising 2% 1-propanol, 100-mM KH_2_PO_4_(pH 7.5), and 30-mM KNO_3_ were added to each tube. Samples were vacuum-infiltrated for 5 min and incubated in a shaking water bath at 25°C for 30 min in the dark. After incubation, 1 mL aliquot from each sample was transferred to a new tube, followed by the addition of 1 mL of sulphanilamide (1% w/v in 1.5 M HCl) and 1 mL *N*-(1-naphthyl)-ethylenediaminedihydrochloride (0.02% w/v in 0.2 M HCl). The samples were incubated at room temperature for 30 min. The absorbance at 540 nm was measured with a spectrophotometer.

All computational studies were carried out on Intel dual core based Microsoft Windows XP professional workstation.

### Sequence Retrieval, Template Search, Secondary Structure Prediction and Sequence Alignment

A search for rice NR at NCBI database revealed the presence of 48 entries. Amino acid sequence of NR from rice (OsNR, accession no. CAA33817.2) was retrieved from the protein sequence database hosted in the National Center for Biotechnology Information (NCBI)^1^. Sequence of rice NR was analyzed for conserved domain at INTERPRO (Apweiler et al., 2001) and gene ontology studies were carried out.

To create a model of the OsNR, at first a BLAST search was performed using the amino acid sequence of OsNR retrieved previously as query. BLASTp (Altschul et al., 1997) was used to identify and retrieve homologous 3D structures by searching the structural database of protein sequence in the protein databank (PDB) (Bernstein et al., 1977)^2^. The protein sequences with PDB-ID 2BIF and 1CNF were identified to have highest percent identity among all the proteins in PDB with OsNR. Secondary structure prediction of OsNR at PROCHECK server (Sharp, 2003) was subsequently carried out.

### Retrieval of the Ligand Structures (Moco, Heme, FAD, Cadmium, NADPH, Nitrate, and Nitrite)

The 3-D structure of organic compounds heme (CID:53627695), flavin adenine dinucleotide (CID: 643975), cadmium (CID:23973), Moco; Molybdenum cofactor (CID: 23304237), NADPH (CID: 52945042), Nitrate (CID: 943) and Nitrite (CID:946) were retrieved at PubChem database (http://pubchem.ncbi.nlm.nih.gov/). Each of these molecules were retrieved in *sdf.file* format from PubChem and converted to *pdb.file* format using Molegro software^3^. Files obtained were used for visualizing 3D structures and for subsequent docking studies.

### Homology Modeling, Model Optimization and Validation

The three dimensional model of NR was predicted by the method of homology modeling. To construct model of the target protein from its amino acid sequence homology modeling is used. Homology modeling exploits the experimental three dimensional structure of a related homologous protein (template) to construct the model. Homology modeling depends on the identification of one or more known protein structures, which share ~30% homology with the query sequence. The basis of this computational technique is that two proteins with similar sequences would adopt similar tertiary structures (Blundell et al., 1987; Sali and Overington, 1994). For modeling of the OsNR, the method involving the satisfaction of the spatial restraints incorporated in the program Modeler 9v of Accelrys Discovery Studio (DS) was used. DS generates a refined 3-D homology model of a protein sequence, utilizing as input its alignment with related structures, which serves as a template. The notion of fulfillment of the spatial constraints derived from the alignment was used by Discovery Studio which is expressed as probability density functions (PDFs). Analytically the PDFs were derived using statistical mechanism and empirically using a database of known protein structures. The spatial restraints and CHARMM energy terms enforcing proper stereochemistry were then joint into an objective function. By optimizing the objective function in Cartesian space the model was obtained finally. A set of 20 models for OsNR were created by Accelrys-DS^4^ using the crystal structure of PDB-ID: 2BIF and 1CNF as a template. The 20 models at various refinement level and library schedules obtained were verified by DS verify protein tool. The best model was selected for energy minimization based on dope score. Energy was minimized by using smart minimizer of DS with maximum 200 steps by steepest descent technique. The best OsNR model obtained after energy minimization was validated using the program PROCHECK^5^. The stereochemical quality of model and protein backbone was inspected by Ramachandran plot analysis, which allows identification of the number of residues with non-ideal torsion angles (Laskowski et al., 2005).

### Molecular Docking Studies of OsNR With Cadmium Metal, Nitrate and Nitrite Alone or in Combination

The three dimensional structure of OsNR obtained above was used for docking studies. Stepwise protein-ligand dockings were performed using Molegro Virtual Docker^3^. The following docking complexes were generated and analyzed (i) OsNR protein backbone with heme cofactor containing Fe at its center, FAD, NADPH and Moco to obtain OsNR (ii) OsNR with nitrate and nitrite to obtain [OsNR-nitrate] and [OsNR-nitrite] complex (iii) [OsNR] complex with cadmium (Cd) metal (iv) [OsNR-nitrate] complex with Cd (v) [OsNR-nitrite] complex with Cd.

### PDB Viewers

Dockings were viewed at Molegro Molecular Viewer and Discovery Studio viewer and useful conclusions drawn after analysis at Pymol^6^ (DeLano, 2002).

### Statistical Analysis

The data were analyzed by a simple one-way variance analysis (ANOVA) and significant differences were compared by *t*-test.

## Results

### Nitrate Reductase Activity in Rice Seedlings Exposed to Cd-Stress

**Figure 2:**
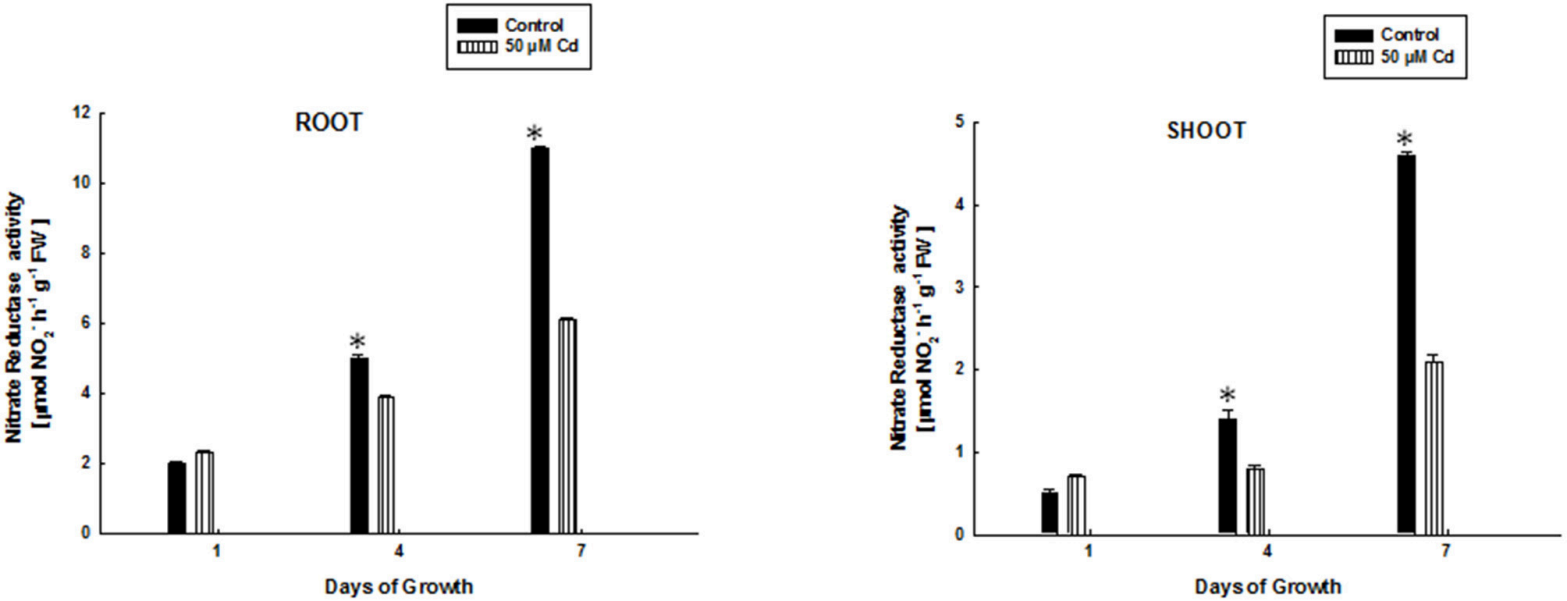
Effect of 50 μM Cd-stress on activity of nitrate reductase enzyme in root and shoot of growing rice seedlings at increasing days of growth. Values are mean of three independent replicates. Error bars indicate S.D. (^*^) indicates values significant at *P* ≤ 0.05.

Figure 2 shows the activity of NR in root and shoot of rice seedlings subjected to 50 μM Cd-stress at 1, 4, and 7 days of growth. It is evident that NR activity is affected by Cd treatment. Any significant difference could not be found in activity of NR between control and Cd treatments in root and shoot of 1 day old rice plants. This could be perhaps because the rice seedlings are likely to be in an adaptive phase during this period. A decreased NR activity by ~1.3–1.8 times at 4 and 7 days in roots of Cd-treated rice plants was noted as compared to control plants. This decrease was ~1.8–2.2 in shoots of Cd-treated rice plants as compared to control at 4 and 7 days of growth. This data suggests an inhibitory effect of Cd on NR activity.

### Sequence Analysis of OsNR

916 amino acid residues were present in the protein sequence of rice NR obtained from NCBI. This sequence showed 89% sequence similarity with 1CNF and 43% with 2BIH.

**Figure 3:**
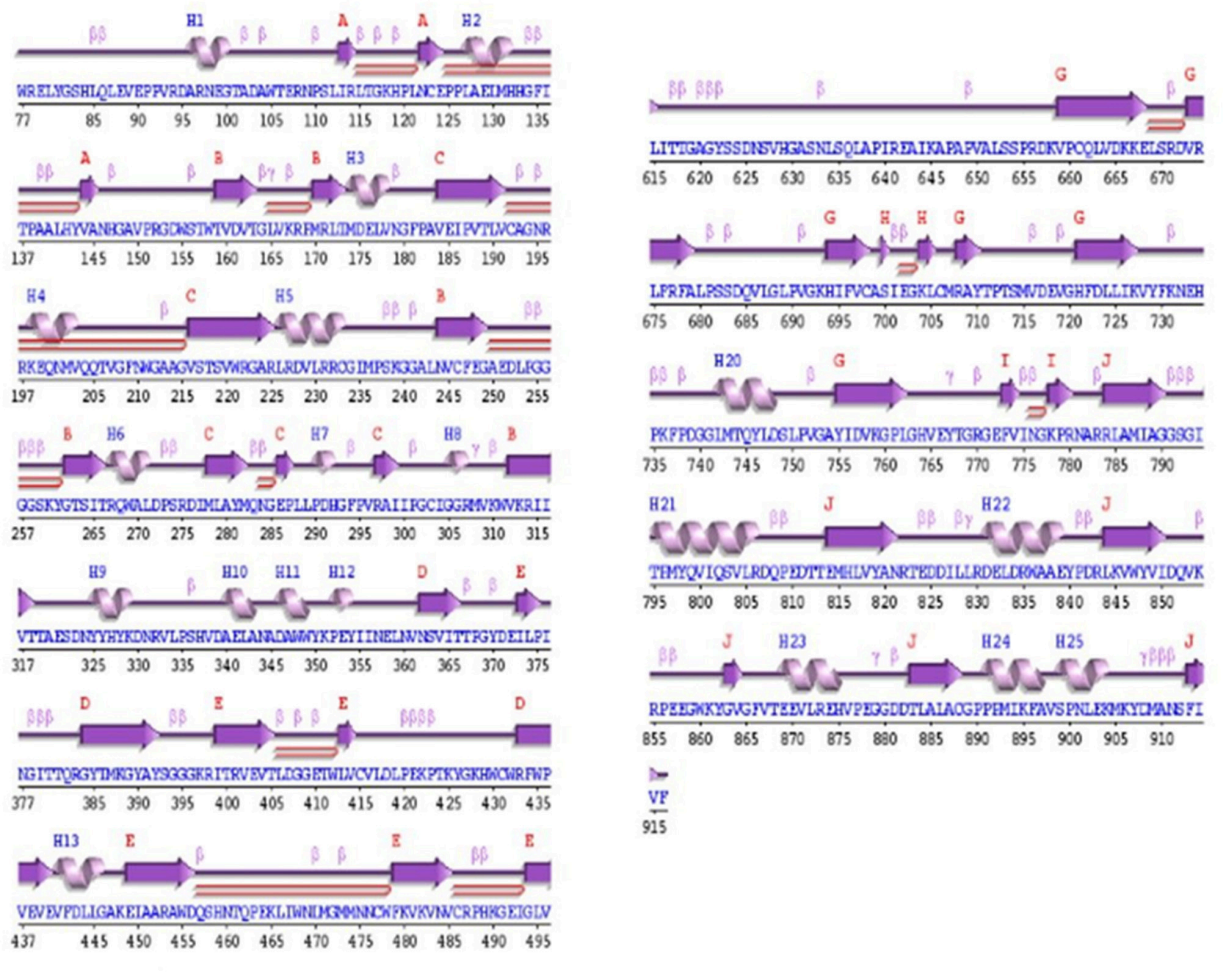
Secondary structure of OsNR showing 10 sheets, 3 beta-alpha units, 14 beta hairpins, 10 beta bulges, 42 strands, 25 helixes, 12 helix-helix turns, 105 beta turns, and 10 gamma turns.

The secondary structure analysis of OsNR was done at PROCHECK and showed that the structure contains 10 sheets, 3 beta-alpha units, 14 beta hairpins, 10 beta bulges, 42 strands, 25 helixes, 12 helix-helix turns, 105 beta turns and 10 gamma turns (Figure 3).

INTERPRO analysis showed that OsNR contains IIPR000572, IPR001199, IPR001433, IPR001709, IPR001834, IPR005066, IPR008333, IPR008335, IPR012137, IPR014756, IPR017927, IPR017938, IPR018506 and IPR022407 which represent molybdopterin binding domain, metal coordination site, dimerization interface, Cytochrome b5-like Heme/Steroid binding domain, FAD binding pocket, and NAD binding pocket motifs.

### Model Prediction, Optimization, and Evaluation

**Figure 4:**
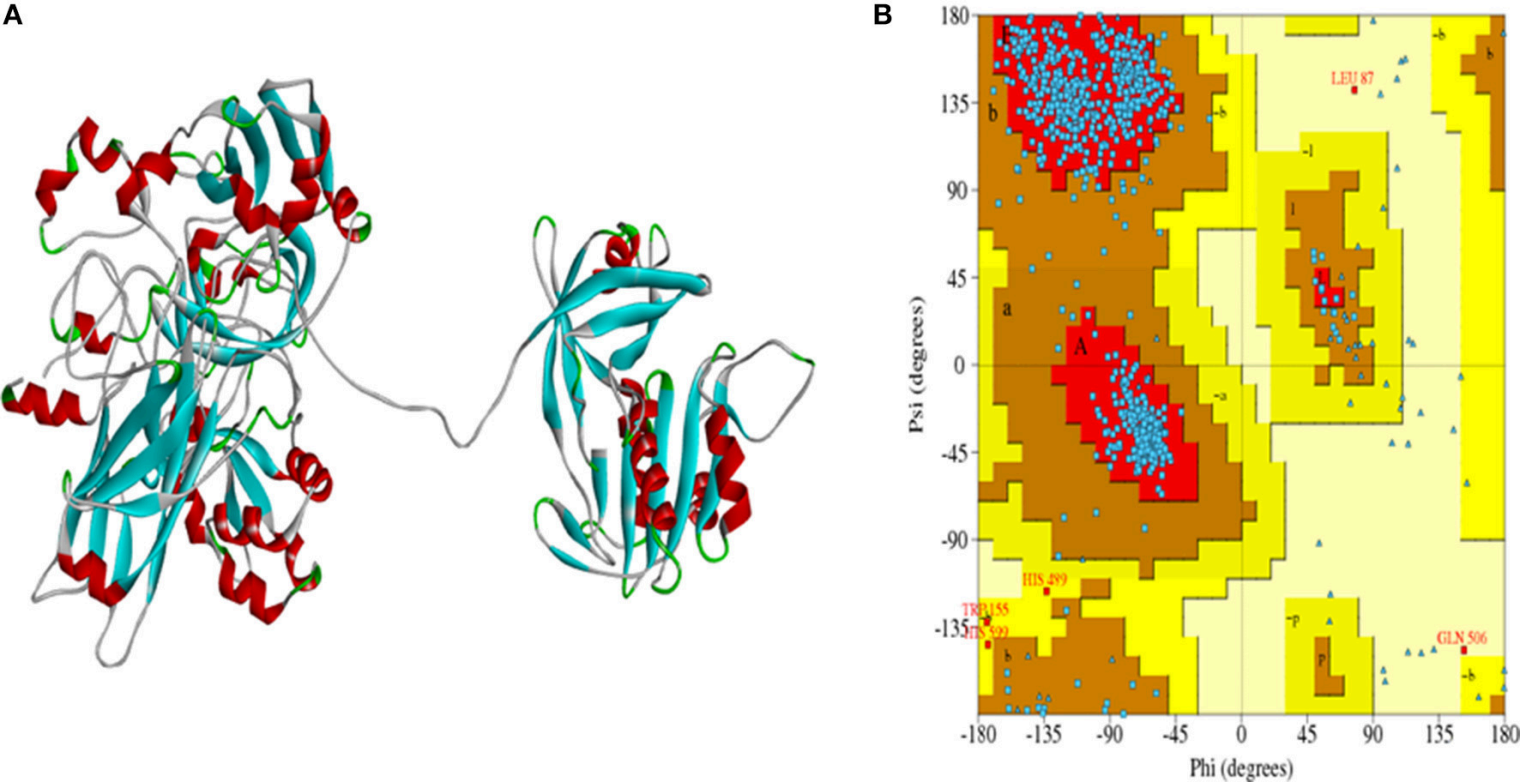
**(A)** Model of OsNR enzyme, **(B)** Ramachandran plot showing 99.7% residues in allowed region and 0.3% residues in disallowed region.

Discovery Studio was used to construct the homology model of rice NR using 1CNF and 2BIH as template. Total 20 models were obtained out of which the best model was selected which had energy −17542.34 kJ/mol. The selected model had high stereo chemical quality as assessed by Ramachandran plot which showed 99.7% residues in allowed regions and 0.3% residues in disallowed regions (Figures 4A,B). The OsNR model obtained by energy minimization was of high-quality.

### Docking and Interaction Analysis

#### Docking of a 3-D Model of Rice NR With Moco, Heme, FAD and NADPH

**Figure 5:**
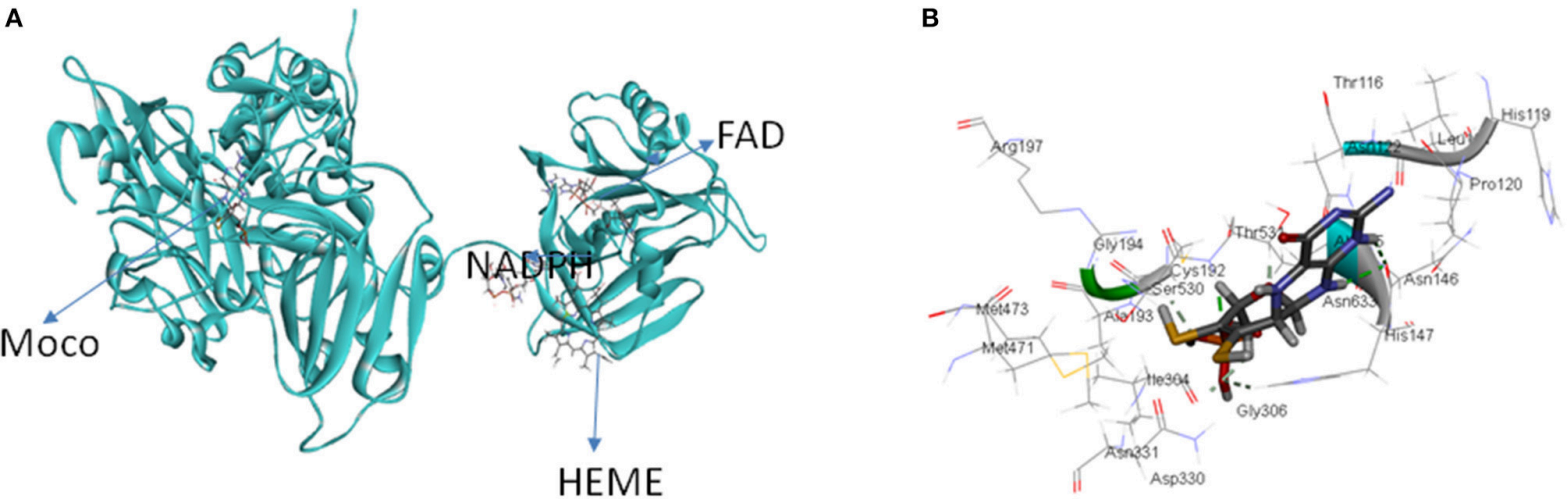
**(A)** OsNR enzyme containing Moco Molybdenum cofactor, heme porphyrin, NADPH (nicotinamide adenine dinucleotide phosphate), and FAD (flavine adenine dinucleotide) ligands. **(B)** OsNR enzyme with conserved amino acid residues at the Moco domain.

Table 1 lists the accession no., molecular weight and IUPAC names of the ligands: Moco, heme, FAD and NADPH obtained from PubChem. These ligands were used for docking with the predicted model of OsNR. OsNR possess a heme, Moco, FAD and NADPH cavities lined with amino acid residues Arg529, Thr531, Asn122, Asp330, His147, Asn146, Ala709, Phe916, Cys888, Gly790, Ser792, Pro796 etc. Ser 700, and Tyr 746 are playing a key role in accommodation of heme inside the NR enzyme. The [OsNR] complex is shown as Figure 5A. As reported by Fischer et al. (2005), our results also revealed that Ala, Gly, Cys, Arg, Hys, Asn, and Ser etc. residues are present at Moco domain. We have also checked for the conserved residues and domains, as per reported by (Fischer et al., 2005), residues Arg79, Arg134, Trp-148 are conserved in our structure, only the positions differ by 10 amino acids (Figure 5B).

**Table 1:** Summary of the ligands used for docking with rice Os-NR as obtained at PubChem database online (http://pubchem.ncbi.nlm.nih.gov/).

**Ligand**	**IUPAC name**	**Chemical identification number (CID)**	**Molecular weight**
Heme	3-[18-(2-carboxylatoethyl)-8,13-bis(ethenyl)-3,7,12, 17-tetramethyl-23H-porphyrin-21-id-2-yl]propanoate;iron(3+); hydrochloride	53627695	651.9402
FAD	[[(2R,3S,4R,5R)-5-(6-aminopurin-9-yl)-3,4-dihydroxyoxolan-2-yl]methoxy-hydroxyphosphoryl] [(2R,3S,4S)-5-(7,8-dimethyl-2,4-dioxobenzo[g]pteridin-10-yl)-2,3,4-trihydroxypentyl] hydrogen phosphate	643975	785.557
Moco	2-amino-4-oxo-8-(phosphonooxymethyl)-1,5,5a,8,9a,10-hexahydropyrano[3,2-g]pteridine-6,7-dithiolate;dioxomolybdenum (2+)	23304237	521.277
NADPH	tetrasodium;[(2R,3R,4R,5R)-2-(6-aminopurin-9-yl)-5-[[[[(2R,3S,4R,5R)-5-(3-carbamoyl-4H-pyridin-1-yl)-3,4-dihydroxyoxolan-2-yl]methoxy-oxidophosphoryl]oxy-oxidophosphoryl]oxymethyl]-4-hydroxyoxolan-3-yl] phosphate	52945042	833.351
Cadmium	Cadmium	23973	112.4110
Nitrate	Nitrate	943	62.004
Nitrite	Nitrite	946	46.005

#### Docking of [OsNR] Complex With Nitrate and Nitrite

**Figure 6:**
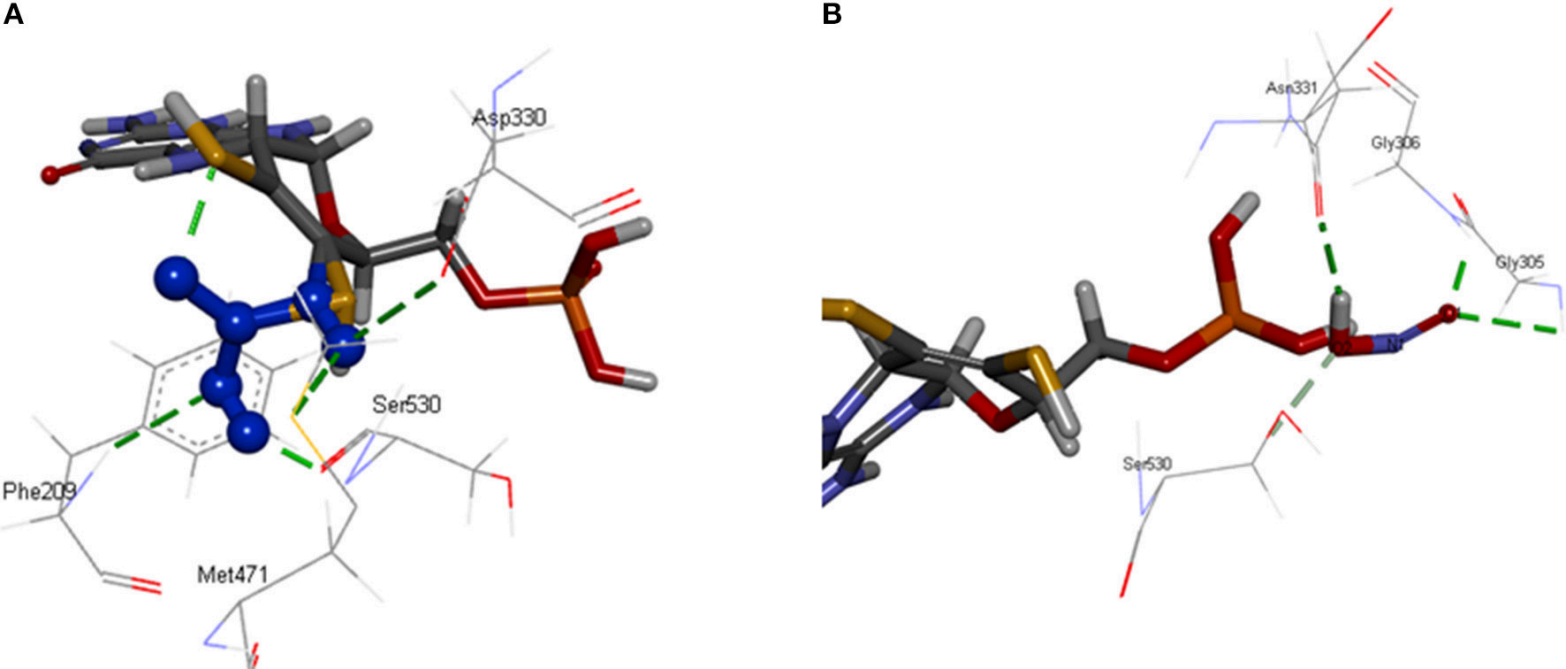
Interaction of OsNR with **(A)** nitrate; **(B)** nitrite.

[OsNR] complex docked with nitrate resulted in [OsNR-nitrate] complex shown as Figure 6A. Many strong H-bonds were formed between OsNR and nitrate with nitrate in the substrate binding cavity formed by amino acid residues Arg197, Arg529, Asp330, Gly208, Met471, Met473, Phe209, Ser530, Thr531, and Val207 (Table 2).

**Table 2:** Complexes [OsNR-nitrate], [OsNR-Cd], [OsNR-nitrite], [OsNR-nitrate-Cd], [OsNR-nitrite-Cd] showing E-pair energy and amino acid residues involved.

**Complexes**	**OsNR-nitrate**	**OsNR-Cd**	**OsNR-nitrite**	**OsNR-nitrate-Cd**	**OsNR-nitrite-Cd**
Total energy of the complex (Kcal/J)	−19282.01	−18336.31	−17885.05	−19760.69	−18746.21
Residues involved	**Nitrate:** Arg197, Arg529, Asp330, Met471, Met473, Thr531, Ser530, Gly208, Phe209, Val207	**Cd:** Arg197, Arg529, Asp330, Met471, Met473, Thr531, Ser530, Asn331, His147	**Nitrite:** Ala193, Ala631, Asn633, Gly305, Met473, Val191, Ser530, Asn331, Gly306, Cys192, Ile304	**Cd:** Ala320,Asp323, Glu321, Ser322, Tyr328 **Nitrate:** Arg307, Asn331, Asp330, Gly262, Gly306, His147, His327, Ile355, Thr263, Tyr261, Tyr326	**Cd:** Arg197, Arg529, Asn331, Asp330, His147, Met473, Ser530, Thr531 **Nitrite:** Ala193, Ala631, Asn633, Gly305, Met473, Val191, Ser530, Asn331, Gly306, Cys192, Ile304

These residues are playing important role in NR enzyme activity. Nitrate is held in the substrate binding cavity of OsNR with the help of four strong H-bonds. Bonds are formed between Phe209-O3, Asp330-OD1, Met471-H1, MOC1-O2, and Ser530-H2 of OsNR and nitrate with bond length 2.83, 2.66, 2.651, and 2.19 Å respectively. No significant structural changes in OsNR after docking with nitrate could be noted as it is a small molecule.

[OsNR] complex docked with nitrite resulted in [OsNR-nitrite] complex shown as Figure 6B. Many strong H-bonds were formed between OsNR and nitrite involving amino acid residues Ala193, Ala631, Asn331, Asn633, Cys192, Gly305, Gly306, Ile304, Met473, Ser530, and Val191 (Table 2). Nitrite is held in the binding cavity of OsNR with the help of four strong H-bonds. Bonds are formed between Gly305-O1, Gly306-O1, H1- Asn331. and Ser530-O2 of OsNR and nitrite with bond length 2.75, 1.81, 2.02, and 2.38 Å respectively. No significant structural changes in OsNR was found after docking with nitrite.

Nitrate and nitrite interactions with OsNR reveal that amino acid residues Met473 and Ser530 are common in both interactions suggesting their binding sites to be in near vicinity (Table 2).

#### Docking of [OsNR] Complex With Cadmium Alone

**Figure 7:**
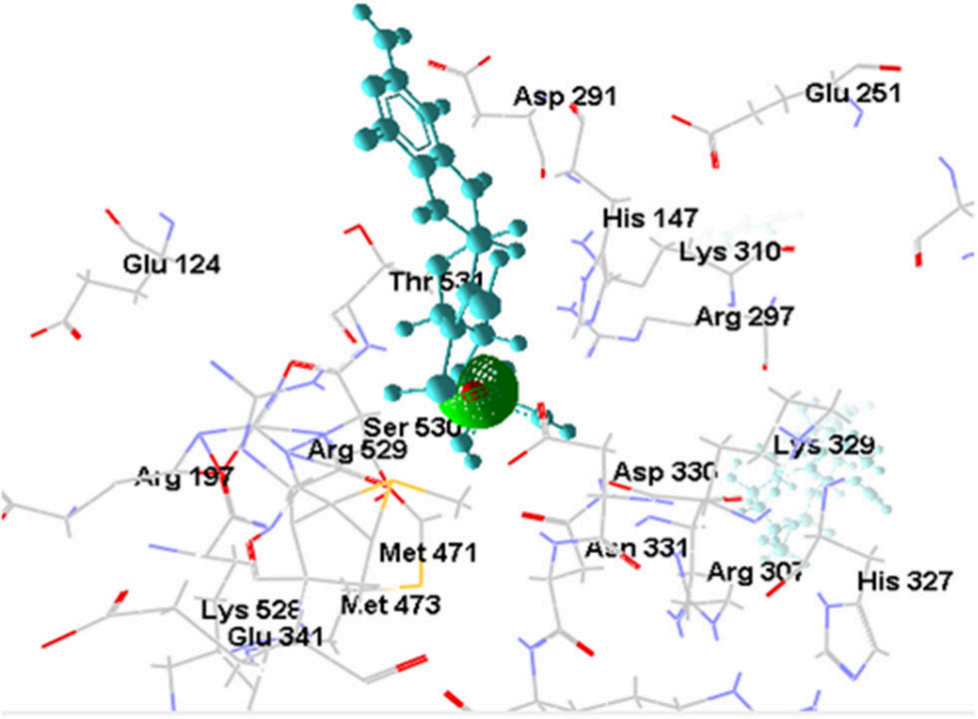
Interaction of OsNR with cadmium.

[OsNR-Cd] complex was formed as a result of docking of cadmium with [OsNR] complex shown as Figure 7. Cd binded in a cavity containing amino acids Arg197, Arg529, Asn331, Asp330, His147, Met471, Met473, Ser530, and Thr531 (Table 2). Electrostatic interaction of 2.17053 Å length with energy −17.6176 Kcal/J and 1.99015 Å length with energy −20.75 Kcal/J is present between OsNR and Cd in [OsNR-Cd] complex.

By comparing the amino acid residues involved in Cd binding alone and amino acid residues involved in nitrate as well as nitrite binding individually, it was found that Cd had 7 amino acid residues common with that of nitrate binding (Arg197, Arg529, Asp330, Met471, Met473, Thr531, Ser530) and three amino acid residues common with that of nitrite binding (Asn331, Met473, Ser530). These results suggest that binding sites of Cd, nitrate and nitrite are in vicinity in the Moco containing subunit of the OsNR.

#### Docking of [OsNR-nitrate] Complex With Cadmium

**Figure 8:**
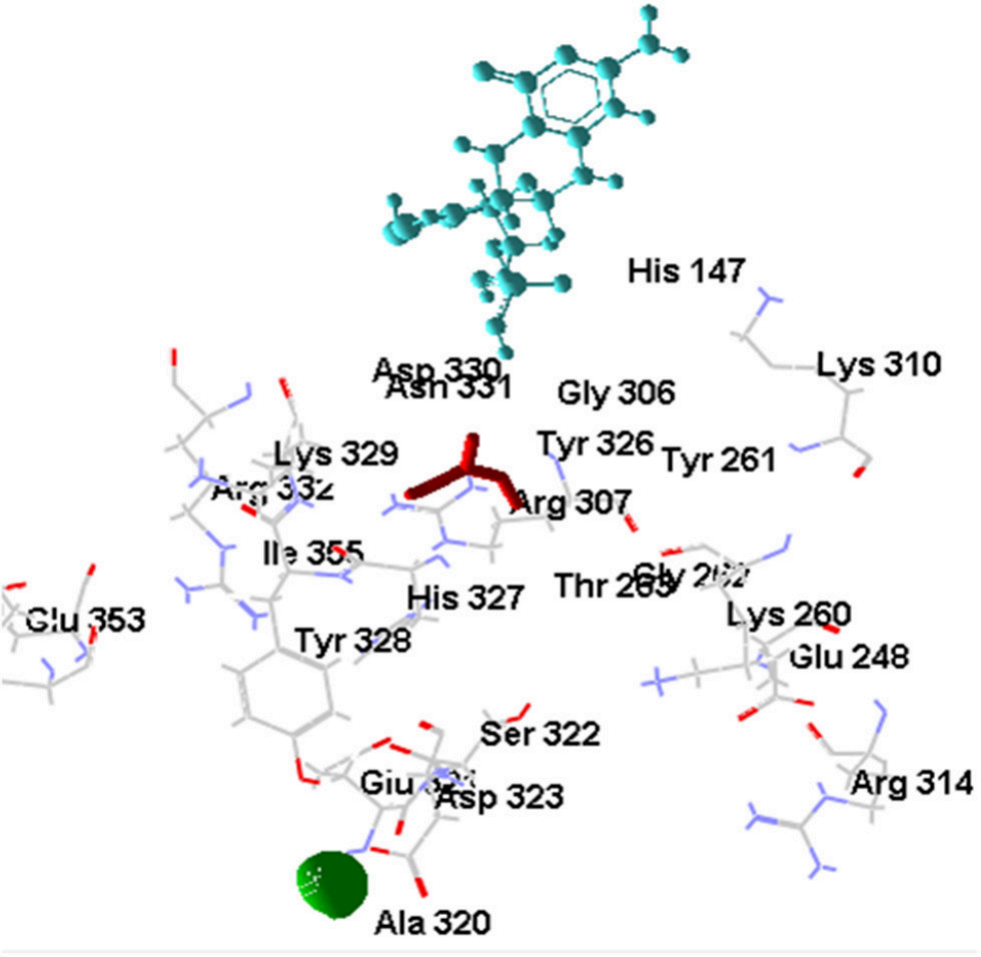
Interaction of OsNR with cadmium and nitrate.

The [OsNR-Nitrate-Cd] complex is shown in Figure 8. Binding of [OsNR-Nitrate] complex to cadmium exposed similar binding positions for both nitrate and cadmium. The residues involved in interactions with nitrate are Arg307, Asn331, Asp330, Gly262, Gly306, His147, His327, Ile355, Thr263, Tyr261, Tyr326. The residues involved in interactions with Cd are Ala320, Asp323, Glu321, Ser322, and Tyr328. Electrostatic interaction of 2.06829 Å length with energy −19.40 Kcal/J and 3.45754 Å length with energy −6.94293 Kcal/J is present between OsNR-nitrate complex and Cd in [OsNR-Nitrate-Cd] complex.

Nitrate binding shows one common amino acid residue with its original binding site while interacting alone (Asp330). While Cd has no common amino acid residues with its original binding site (Table 2).

#### Docking of [OsNR-nitrite] Complex With Cadmium

**Figure 9:**
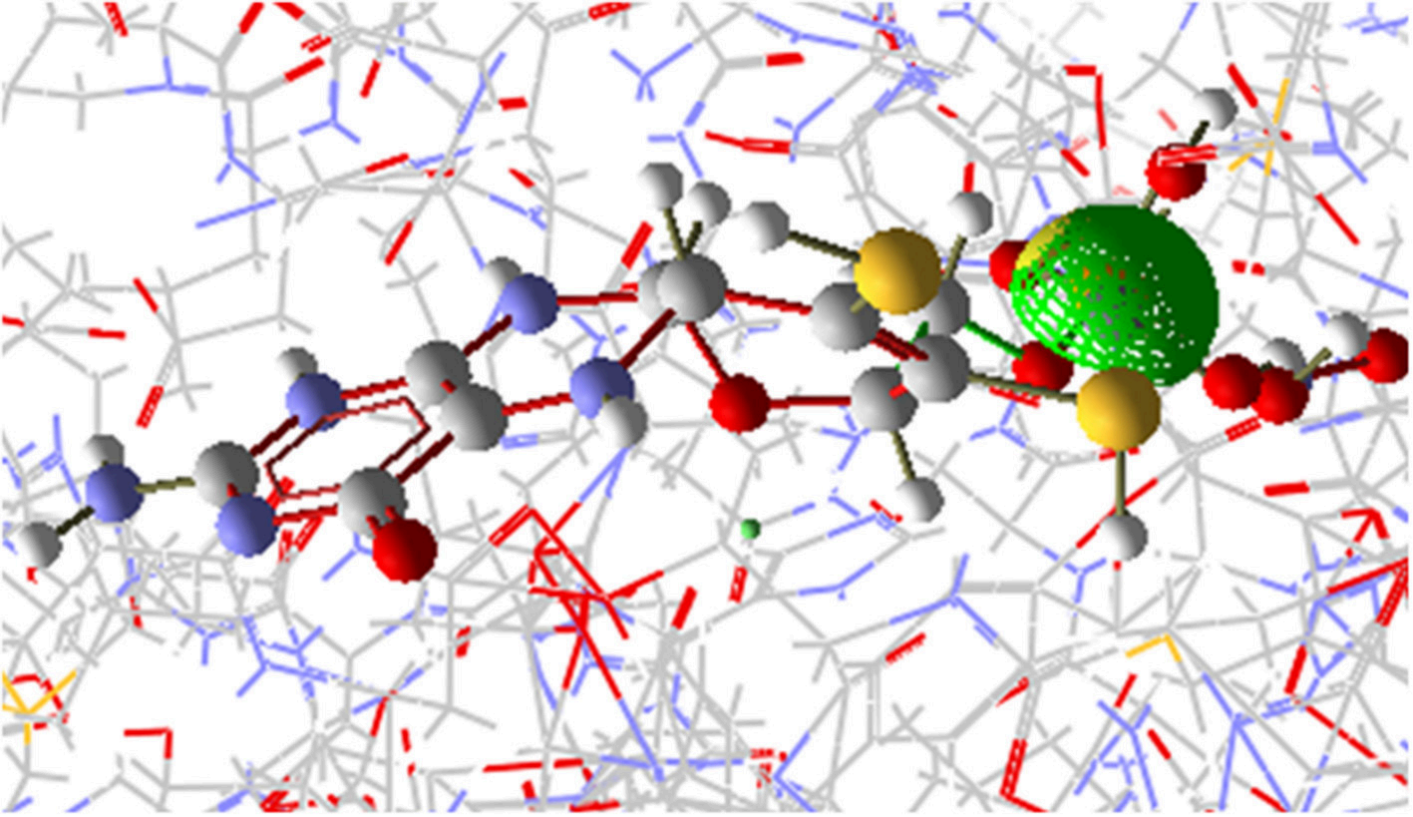
Interaction of OsNR with cadmium and nitrite.

The [OsNR-Nitrite-Cd] complex is seen as Figure 9. The residues involve in interactions with nitrite are Ala193, Ala631, Asn633, Gly305, Met473, Val191, Ser530, Asn331, Gly306, Cys192, Ile304. Amino acid residues interacting with Cd are Arg197, Arg529, Asn331, Asp330, His147, Met473, Ser530 and Thr531. Asn331, Met473, Ser530 are common residues for Cd and nitrite in [OsNR-Nitrite-Cd] complex. These results suggest that Cd binds to the same site in [OsNR-nitrite-Cd] complex as compared to its binding site in [OsNR-Cd] complex and it is competing with nitrite for the active site of the enzyme in [OsNR-Nitrite-Cd] complex.

## Discussion

Nitric oxide, a signaling molecule, is of prime importance inside the plant system. It plays role in many plant processes such as programmed cell death, stomatal closure, growth, germination, signaling, biotic and abiotic stress response (Wendehenne, 2011; Mur et al., 2012; Sanz-Luque et al., 2015; Farnese et al., 2016). Many results point toward enzyme NR as an important enzyme for nitric oxide production (Gupta et al., 2011; Mur et al., 2013). NR is a molybdoenzyme, all eukaryotic enzyme containing molybdenum have a molybdenum (Mo) atom attached to two sulfur atoms (ene-dithiolate) of molybdopterin (a pyranopterin derivative). The molybdopterin contains a highly conserved structural core which is present in nearly all Mo containing enzymes except for nitrogenase (Fischer et al., 2005). Rice *(Oryza sativa* L.) is a worldwide significant crop with extensive studies on effects of different stresses on its growth and development. In the wet-lab studies it was observed that exogenous application of cadmium (Cd) caused decreased NR activity in rice seedlings as compared to control plants. With the knowledge of complete rice genome (Ohyanagi et al., 2006) it is expected that the 3-D structures of important rice proteins would be available in the database online. Surprisingly, no crystallographic structure could be obtained or is reported for rice NR. With the help of homology modeling, 3-D structure of rice-NR (OsNR) is modeled in this study. Binding of OsNR with nitrate, nitrite and cadmium metal *in silico* has been explored. A detailed study of interactions between enzyme-substrate complex and cadmium and the alterations in the bonds formed in presence of cadmium is carried out *in silico*. The bonds formed between the enzyme-substrate complex, enzyme-cadmium and differences in interactions in presence of cadmium has been studied in detail *in silico*. This is the first report to show structure of rice NR *in silico* based on previous literature (Dias et al., 1999; Campbell, 2001; Fischer et al., 2005; Seenivasagan et al., 2016; Chamizo-Ampudia et al., 2017). Many earlier reports have suggested a negative impact of heavy metal Cd on NR activity (Rai et al., 1998; Gouia et al., 2000; Irfan et al., 2014). Though there is a lot of wet lab work on the effect of cadmium toxicity on NR activity in plants, still the mechanism underneath has not been explored much. Through our *in-silico* work we have tried to investigate the reason behind negative impact of Cd on NR activity for the first time. OsNR structure was modeled using homology modeling and the best model was selected on the basis of energy. Ramachandran plot of the model shows 99.7% residues in the allowed region which shows that the model is of good quality, reliable and robust. Moco, FAD, NADPH and Heme are required for a functional NR enzyme hence all these ligands were docked with OsNR to obtain functional enzyme. Docking of functional OsNR with nitrate and nitrite showed that both are binding on the Moco containing site, which is also the active site of the enzyme (Fischer et al., 2005). Docking of OsNR with nitrate and nitrite resulted in several strong H-bonds between enzyme OsNR and nitrate as well as nitrite. In this study Met473 and Ser530 were found to be common binding residues for both nitrate and nitrite suggesting thereby that nitrite may act as a competitive inhibitor of NR. Docking of OsNR with Cd showed interesting results, it was found that Arg197, Arg529, Asp330, Met471, Met473, Ser530, Thr531 were playing important role in binding of Cd to OsNR. Comparison of the amino acid residues of both nitrate and Cd binding revealed seven amino acids *viz*: Arg197, Arg529, Asp330, Met471, Met473, Ser530, Thr531 to be common. However Asn331, Met473 and Ser530 were common to binding of nitrite and Cd only. It can therefore be concluded that Cd is binding to a similar site on OsNR as that of nitrate and nitrite. However when plants are exposed to Cd-stress, both nitrate, nitrite and Cd will be present in a plant's internal environment, hence nitrate, nitrite and Cd were docked with OsNR individually. Results were very interesting and suggest changes in the original binding sites of both Cd and nitrate when they are interacting alone with OsNR. Nitrate binding shows one common amino acid residue with its original binding site while interacting alone (Asp330) in [OsNR-nitrate-Cd]. Whereas Cd has no common amino acid residue with its original binding site. It can be inferred that in [OsNR-nitrate-Cd] complex, nitrate is binding near its original binding position while interacting alone with OsNR. This may result in a low enzyme activity due to lack of proper binding of the substrate nitrate. Cd, which is binding to an entirely different site in [OsNR-nitrate-Cd] complex as compared to its original binding site in [OsNR-Cd] complex, may also bring some conformational changes in the enzyme structure thus resulting in a decreased enzyme activity. Also in the binding of OsNR with both nitrite and Cd it was found that both nitrite and Cd were binding on the Moco domain. So it might be assumed that both of them are competing with each other for the active site, hence reducing the activity of the enzyme. There are also some changes in the amino acid residues of nitrite and Cd binding in [OsNR-nitrite-Cd] complex as compared to their residues in OsNR-nitrite and OsNR-Cd complexes. In presence of both nitrate and Cd with OsNR, nitrate shifted from its original position which might lead to a decreased NR activity as the substrate is not binding to the active site of the molecule. Cd, binding to an entirely new position in OsNR-nitrate-Cd docking as compared to its original position in OsNR-Cd complex, might have caused some structural changes in the enzyme. Both these occurrences might result in an altered or decreased NR activity. Competition between both Cd and nitrite for the same binding site in [OsNR-nitrite-Cd] complex might also lead to a decrease in NR activity. These results may establish a possible correlation between decrease in NR activity and Cd toxicity in plants for the first time.

## Conclusion

Interactions involved with the binding of cadmium with rice NR in presence and absence of nitrate or nitrite was analyzed *in silico*. Docking of OsNR with nitrate and nitrite resulted in several strong H-bonds. Met473 and Ser530 were found to be common binding residues for both nitrate and nitrite suggesting thereby that nitrite may act as a competitive inhibitor of NR. Amino acid residues: Arg197, Arg529, Asp330, Met471, Met473, Ser530, Thr531 were playing important role in binding of Cd to OsNR. Seven amino acids: Arg-197, Arg- 529, Asp-330, Met-471, Met-473, Ser- 530, Thr-531 were found to be common in binding of Cd and nitrate with OsNR. Amino acid residues Asn331, Met473 and Ser530 were found to be common in nitrite and Cd binding. It can probably be concluded that Cd is binding to a similar site in OsNR as that of nitrate and nitrite. In presence of both nitrate and Cd with OsNR, nitrate shifted from its original position which might lead to a decreased NR activity as the substrate is not binding to the active site of the molecule. It is the first report in rice which well correlates with the wet-lab results where activity of NR decreased under Cd-stress perhaps due to altered binding dynamics involving different amino acid residues.

## Author Contributions

PS and IS have performed all the wet lab and computational studies. PS, IS, and KS have prepared and edited the manuscript jointly.

### Conflict of Interest Statement

The authors declare that the research was conducted in the absence of any commercial or financial relationships that could be construed as a potential conflict of interest.

## Footnotes

^1^http://www.ncbi.nlm.nih.gov

^2^http://www.rcsb.org

^3^https://molegrovirtualdocker.weebly.com/

^4^http://www.3dsbiovia.com/resource-center/downloads/updates/discovery-studio/dstudio35/latest.html

^5^http://servicesn.mbi.ucla.edu/SAVES/

^6^http://pymol.sourceforge.net/index.html

## Abbreviations

Cd: cadmium
nR: Nitrate reductase
NOFNiR: nO-forming nitrite reductase
Mo: molybdenum
Moco: molybdenum cofactor
FAD: flavin adenine nucleotide
SO: sulfite oxidase
Fe: iron
NAD(P)H: nicotinamide adenine dinucleotide phosphate
OsNR: rice-nitrate reductase
DS: discovery studio.
